# Estimation of the age of human semen stains by attenuated total reflection Fourier transform infrared spectroscopy: a preliminary study

**DOI:** 10.1080/20961790.2019.1642567

**Published:** 2019-09-09

**Authors:** Shuai Zha, Xin Wei, Ruoxi Fang, Qi Wang, Hancheng Lin, Kai Zhang, Haohui Zhang, Ruina Liu, Zhouru Li, Ping Huang, Zhenyuan Wang

**Affiliations:** a Department of Forensic Pathology, Xi'an Jiaotong University School of Medicine, Xi’an, China;; b Department of Forensic Medicine, Chongqing Medical University, Chongqing, China;; c Department of Forensic Medicine, Xuzhou Medical University, Xuzhou, China;; d Department of Forensic Pathology, Academy of Forensic Science, Shanghai, China

**Keywords:** Forensic sciences, forensic medicine, semen stain, age estimation, spectroscopy, Fourier transform infrared, chemometrics

## Abstract

Semen stain is one of the most important biological evidence at sexual crime scenes. Age estimation of human semen stains plays an important role in forensic work, and it is rarely studied due to lack of well-established methods. In this study, the technique called attenuated total reflection Fourier transform infrared spectroscopy (ATR-FTIR) coupled with advanced chemometric methods was employed to determine the age of semen stains on three different substrates: glass slides, tissues and fabric made of regenerated cellulose fibres up to 6 d. Partial least squares regression (PLSR) was used in conjunction with spectral analysis for age estimation, and the results generated high *R*^2^ values (cross-validation: 0.81, external validation: 0.74) but a narrow margin of error for root mean square error (RMSE) (RMSE of cross-validation: 0.77 d, RMSE of prediction: 1.02 d). Additionally, our results indicated the robustness of PLSR model was not weaken by the influence of different substrates in this study. Our results indicate that ATR-FTIR, combined with chemometric methods, shows great potential as a convenient and efficient tool for age estimation of semen stains. Moreover, the method could be applied to routine forensic investigations in the future.

## Introduction

Body fluids, such as blood, semen, vaginal secretion and saliva, are typical specimens collected as evidence at crime scenes. Semen is the most reliable marker in rape, sodomy and other forensic cases. It can be used to confirm sexual assault and identify suspects [1, 2]. In addition, it can be used to estimate the time frame when a crime happened. The rate of crime has increased rapidly over the years in China [3]. Forensic analysis is confronted with great challenges. For challenging cases, such as when the victims are mentally handicapped, no eyewitnesses were present, or the victims do not survive, determining the time period of the crime involves indirectly estimating the wound age or postmortem interval (PMI).

In forensic investigations, numerous methods of semen identification have been investigated, including presumptive and confirmatory tests. Presumptive assays, such as seminal acid phosphatase, and confirmatory tests, such as the Christmas tree stain for the observation of spermatozoa, are widely used in routine forensic work. Moreover, protein-based immunologic assays, such as the detection of semenogelin antigen and prostate-specific antigen (PSA), as well as other DNA- or RNA-based assays, are performed in forensic laboratories [4]. UV–vis, Fourier transform infrared spectroscopy (FTIR) and Raman spectroscopy have also been used for identification and discrimination of body fluids [5]. Interestingly, semen samples can be used to predict human age using genetic analysis based on DNA methylation [6]. However, few studies have estimated the age of semen stains, which can play an important role in forensic investigations. If the age of a semen stain was known, investigators could potentially verify alibis, identify suspects, determine the time of crimes and indirectly estimate the PMI.

Infrared spectroscopy is a rapidly developing technology that has been widely used in forensic analysis. It is a fast and nondestructive technique that requires minimal sample consumption. FTIR is a valuable detection tool with high sensitivity and the ability to detect changes in macromolecules in biological materials [7, 8]. A large number of studies have applied FTIR to forensic casework to differentiate materials, such as paper, paint, coating, hair and propellant in explosive devices [9–13]. Moreover, FTIR has been used for the analysis, detection and identification of body fluids and biological tissues [14, 15]. In our previous work, satisfactory results were achieved using attenuated total reflection-FTIR (ATR-FTIR) for the analysis of time-dependent changes in biological tissues, combining with chemometric methods which further improved the accuracy of the results [16–20]. In the field of semen research, FTIR has been used to characterize human sperm in clinical settings [21]. In addition, it has been used for rapid detection of semen stains on various substrates [1, 22]. It offers numerous advantages compared to DNA-based methods, which lead to sample destruction [1]. Moreover, FTIR has been used to evaluate changes in bloodstains during the time since deposition (TSD), and several noteworthy studies have used spectroscopic methods to estimate the age of bloodstains [18, 23, 24]. Recent advances have paved a new direction for determining the time at which crimes occur [16]. Thus, semen stain age analysis via FTIR can provide key information in determining the time of a crime and for estimating PMI.

In this study, we developed a method to determine the time at which a semen stain was formed using ATR-FTIR. To improve the accuracy and availability of spectral data analysis, chemometrics were employed in our study as a reliable and established method for identifying subtle spectral differences and converting the information into available predictive models. The age of semen stains was predicted, and we believe this method shows great potential for routine forensic analysis.

## Materials and methods

### Sample preparation

Samples of semen were collected from eight healthy male volunteers (ages from 22 to 30), of which two volunteers were chosen for external validation set randomly. The volunteers signed an informed consent agreement and were informed of the project objectives. In trying to simulate realistic semen stain at a crime scene, three different substrates were used: glass slides, tissues and fabric made of regenerated cellulose fibres. For the calibration set, each semen sample from six donors was applied dropwise onto each substrate, and samples were collected at 0.5, 1, 1.5, 2, 2.5, 3, 3.5, 4, 4.5, 5, 5.5 and 6 d. The substrates were kept at constant temperature and humidity (ambient temperature of (25 ± 1)°C and relative humidity of (45 ± 5)%). Additionally, samples from the other two donors were used as an external validation set. The samples in the external validation set were also collected on each substrate, with the same sampling time points. These samples were kept in a ventilated room with uncontrolled conditions (ambient temperature: 14˚C–28˚C, relative humidity: 35%–80%, approximately).

### Spectral collection and pre-treatment

A Nicolet 5700 FTIR spectrometer (Thermo Fisher Scientific, Waltham, WA, USA) equipped with a diamond crystal ATR and a deuterated triglycine sulphate detector (Thermo Fisher Scientific) was used for spectral obtaining. OMNIC version 8.2 (Thermo Nicolet Analytical Instruments, Madison, WI, USA) is an infrared spectra analysis software package that was employed for spectra data recording and spectral analysis of FTIR. Semen stain samples on glass slides were scraped into a tube and mixed with 10 μL normal saline prior to centrifugation at 3 000 r/min. The supernatant was collected. Stain samples on the other two substrates were wetted by 10 μL of normal saline and centrifuged at 3 000 r/min for 1 min in spin columns with no filter. The liquid was collected in collection tubes.

Three 1 μL drops of each sample were applied to the ATR crystal and dried by a fan. Before each test, the ATR crystal was swabbed with anhydrous ethanol and dried prior to collecting the background. The spectra were collected in the region of 4 000–900 cm^−1^, with 32 scans and resolution of 4 cm^−1^. Each drop was recorded by three repetitive operations, to ensure the repeatability of the method and to reduce the error caused by inhomogeneity of sample dissolution. Totally, 2 592 spectra were collected.

The raw absorbance spectra were pre-processed and analyzed by PLS Toolbox 8.1.1 (Eigenvector Research, Manson, WA, USA) in Matlab R2017a (MathWorks, Natick, MA, USA), including standard normal variate (SNV). Subsequently, second derivatives were used to detect the subtle band components hidden in the broad overlapping components by a 13-point Savitsky–Golay second-derivative function, which can reduce the background signal and light scattering caused by changes in the physical properties of semen stains. The 13-point Savitsky–Golay second-derivative function is also used to enhance the accuracy of subsequent multivariate analysis [25]. Then, mean centre was also employed for preprocessing. All processing was done in the region of 1 800–900 cm^−1^, which is referred to as the “bio-fingerprint” region in spectroscopy. This region contains the most valuable information of biomolecules, such as protein, lipids, nucleic acids and sugars [26].

### Multivariable statistical analysis

Principle component analysis (PCA) and partial least squares regression (PLSR) were used to analyze the spectral data and evaluate the effects of different substrates. Moreover, the PCA and PLSR analyses were used to generate regression models. PCA is a modelling method that extracts a set of correlated variables and converts them into a smaller set of uncorrelated variables called principal components (PCs), which are comprised of information from raw spectra [25]. PCA can decrease the dimension of features, reduce the complexity of computation and seeks for maximum variance, providing a more convenient classification and cluster analysis approach. In this study, to ensure that the substrates do not interfere in spectral analysis and identify outliers, spectral datasets were transformed into two-dimensional score plots by PCA. Outlying samples within the groups were identified as outliers and were excluded. Ultimately, with high leverage values and Q-residuals [27], three abnormal samples were considered outliers and removed prior to regression modelling, of which the percentage was below 2% of the total specimens.

As for PLSR, this high-throughput can construct multiple regression models with linear relationships between variables X and Y [25]. The values of the Y variables, which are associated with the age of semen stains in this study, could be predicted from a large set of X variables (i.e. the matrix of spectral data). To determine the number of latent variables (LVs), which is a crucial step for optimization of the model, leave-one-out cross-validation (CV) was used. With a value of the root mean square error (RMSE) of CV (RMSECV) below 5%, the best number of LVs was selected [28].

For evaluation of the PLSR models, RMSECV and the RMSE of the predications (RMSEP) were used in the internal validation and the external validation, separately, as well as the *R*^2^ values. Our objective was to find a reliable PLSR model with a high *R*^2^ value and a low RMSE value.

## Results and discussion

**Figure 1.**
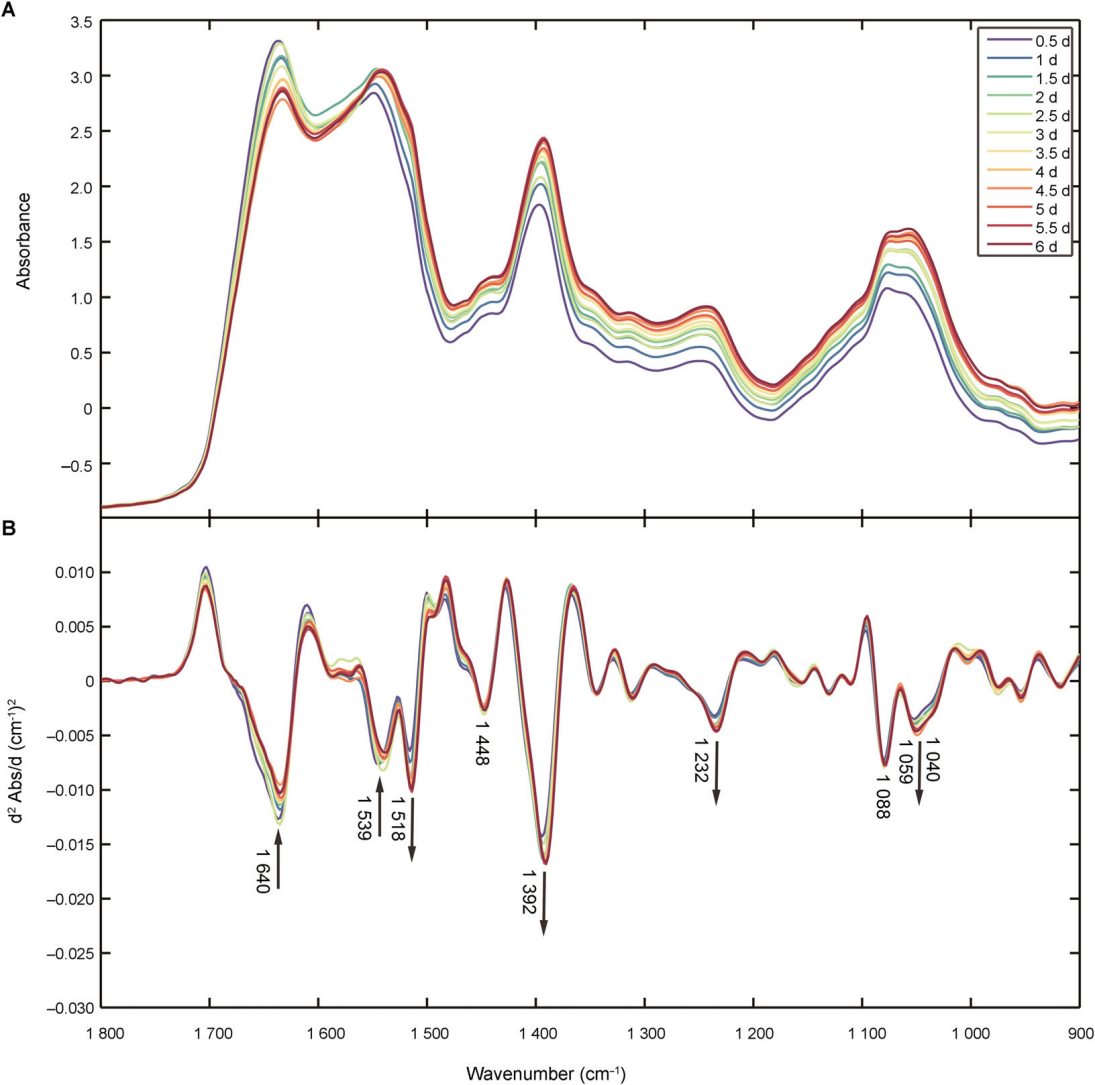
(A) FTIR averaged spectra of semen stains at different time points in the range of 1 800–900 cm^−1^. (B) The spectra of second derivative transformation at different time points in the same range.

Average spectra, in the region of 1 800–900 cm^−1^, for semen stains of different ages from the calibration set are shown in Figure 1(A). Figure 1(B) shows the averaged second derivative transformation of spectra prior to comparison. The peaks at 1 640 cm^−1^ and 1 539 cm^−1^ were attributed to amide I and amide II, respectively. The band at 1 518 cm^−1^ was attributed to tyrosine, while the peaks at 1 448 cm^−1^ were characteristic of asymmetric methyl bends in amino acid side chains of proteins. Another noticeable peak at 1 392 cm^−1^ was attributed to symmetric methyl bends in the amino acid side chains of proteins. The band at 1 059 cm^−1^was attributed to PSA in semen. The lower intensity peaks at 1 232 cm^−1^, 1 088 cm^−1^ and 1 040 cm^−1^ were associated with nucleic acid phosphates, symmetric vibration of phosphates and carbohydrates (glucose, polysaccharides and fructose), respectively (Table 1) [29].

**Table 1.** Major Fourier transform infrared spectroscopy (FTIR) peak component assignment of semen stain.

Frequency (cm^−1^)	Assignment
1 640	Amide I: α-helix
1 539	Amide II: β-sheet
1 518	Tyrosine
1 448	C–H bending vibrations of -CH_2_ and -CH_3_ groups
1 392	Symmetric vibration of COO^−^: fatty acids and polysaccharides
1 232	Asymmetric vibration of PO^2−^
1 088	Symmetric vibration of PO^2−^
1 059–1 040	Sugar moieties within glycoproteins
954	Symmetric C–O stretching from carbohydrates

### Comparison of changes in the spectrum

Over time, the absorption spectra of semen stains changed considerably (Figure 1(B)). The decrease in peak intensity at 1 640 cm^−1^ and 1 539 cm^−1^ could be due to the degradation of proteins and other macromolecules, which occurs in human tissues, blood stains and body fluids over the postmortem time [17–19]. The increase in peak intensity at 1 392 cm^−1^ (representing COO^−^ stretching) may be caused by break of peptide chain and increment of free amino acids. As for the PO^2−^ peaks at 1 232 cm^−1^, the increase in peak intensity may be related to the degradation and rupture of sperm cells. Since DNA strand breaks in the sperm heads could happen in normal sperm after being frozen or other conventional methods of sperm preprocess [30], spermatozoa with DNA strand breaks in semen stains could not be avoided in this study. DNA strand breaks occur as a result of oxidation and other environmental factors, which may ultimately lead to the release of nucleic acids and other free phosphates into seminal plasma.

Nevertheless, it is inaccurate and inefficient to explain the variation in spectra and estimate the age of semen stains based solely on changes in several spectral peaks. Therefore, in order to gain more specific information from the spectral data, regression models of semen stains and multivariate chemometric methods were employed in the following experiments.

**Figure 2.**
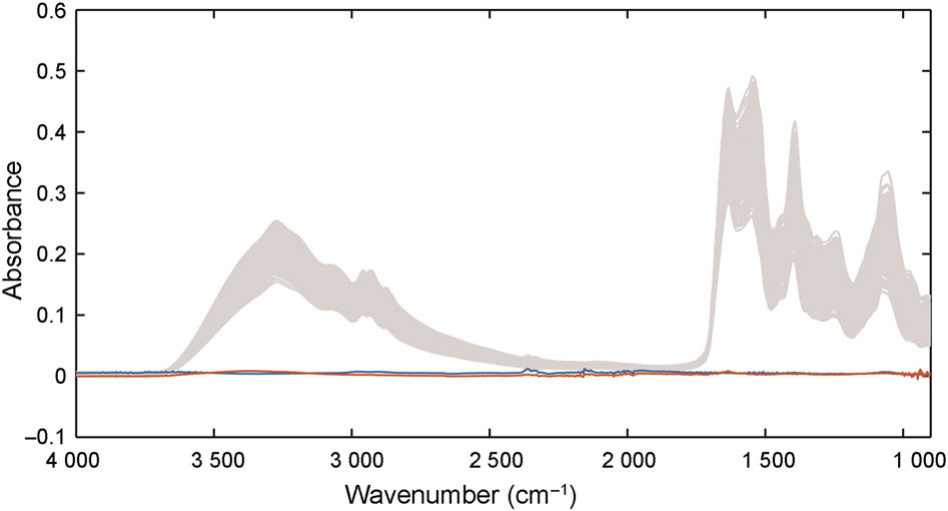
Raw spectra from semen stains (grey lines) and eluent from semen stains on tissues (orange line) and fabric made of regenerated cellulose fibres (blue line).

**Figure 3.**
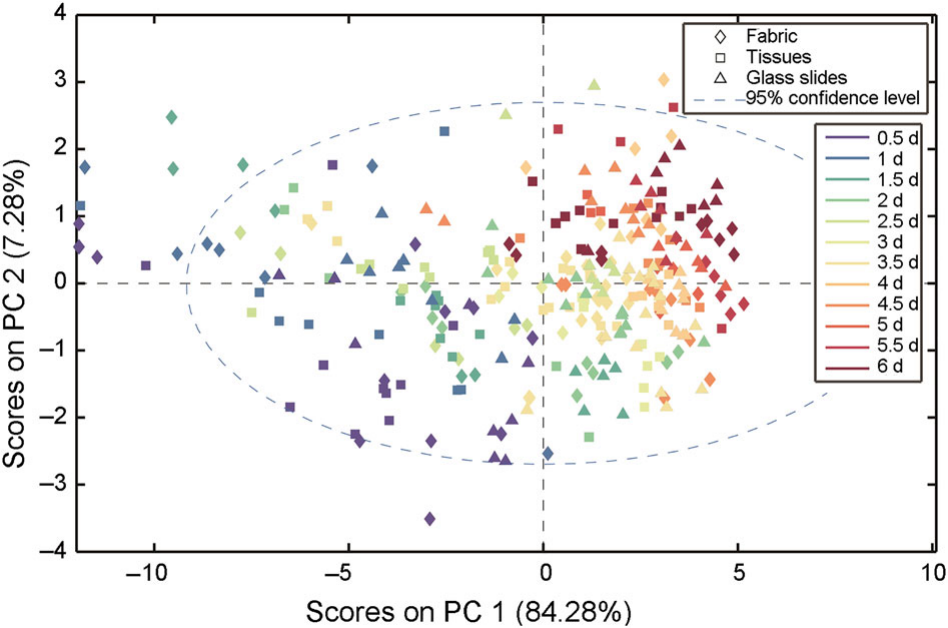
Principle component analysis result of different samples of semen stains on three substrates over 6 d. Complicated overlap indicates small variation between different substrates. PC: Principle component.

The raw spectra of the eluent washed off from the two substrates (tissues and lady panties made of regenerated cellulose fibres) without stains were collected, while the raw spectra obtained from three substrates were also shown in Figure 2, which indicates the various substrates had minimal effects on the analysis of semen stains. The minimum absorption of the semen sample was approximately 0.1, and the maximum absorption of the substrates was only 0.005, which may be due to cellulose and other impurities. Because of the uniformity of synthetic fabric in tissues and polyesters, less semen stains was remained on these fabric than that on other thicker weave carrier such as denim and wool, which could trap some sperm cells during washing [31]. Thus, in this study, centrifugation and saline washing ensured most spermatozoa and other seminal components were removed from the samples. PCA was also employed for the spectra collected from the individual substrates, and the results are shown in Figure 3. Semen stains on three substrates over 6 d were compared, and the two PCs show high overlap, with the explanation of 84.28% variances. This indicates the variation between the three substrates was negligible for this model and the factor of them in establishment of regression models can be irrespective in this study. The results demonstrate that macromolecules in semen stains change over time and are not substrate-dependent. Therefore, the regular variations of the spectra can be used effectively for the subsequent regression models.

### Establishment of the PLSR model for age estimation

**Figure 4.**
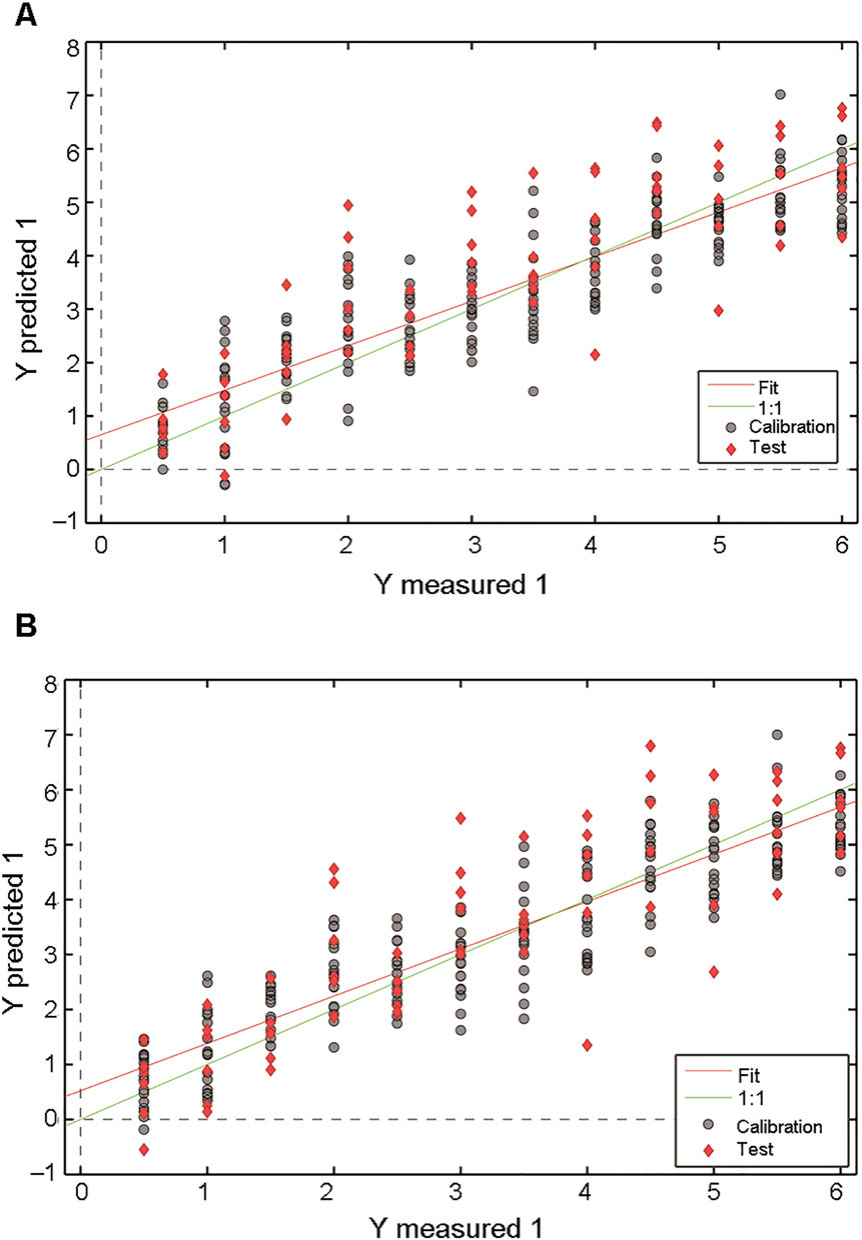
Results from the internal validation and external validation sets by (A) partial least squares regression (PLSR) models and (B) second derivative transformation by PLSR models in 0.5–6 d period. The grey dashed lines are the reference lines corresponding to the perfect external validation.

Both the FTIR spectra and second derivative spectra of the “bio-fingerprint” region were used in the regression models for age estimation of semen stains over 6 d (Table 2). The PLSR model generated mediocre results (Figure 4(A)), with *R*^2^ values of 0.79 and 0.72 for the cross-validation and external validation, respectively, as well as RMSE values of 0.80 d and 1.06 d for internal and external validation, respectively. Figure 4(B) shows the calibration results of the model based on second derivative spectra of the entire “bio-fingerprint” region (spectral range from 1 800 to 900 cm^−1^). High *R*^2^ values (cross-validation: 0.81, external validation: 0.74) and a narrow margin of error for RMSE (RMSECV: 0.77 d, RMSEP: 1.02 d) were achieved, as well as the smaller ratio of RMSEP/RMSECV. Five LVs were adopted in the PLSR model of the filtered spectrum.

**Figure 5.**
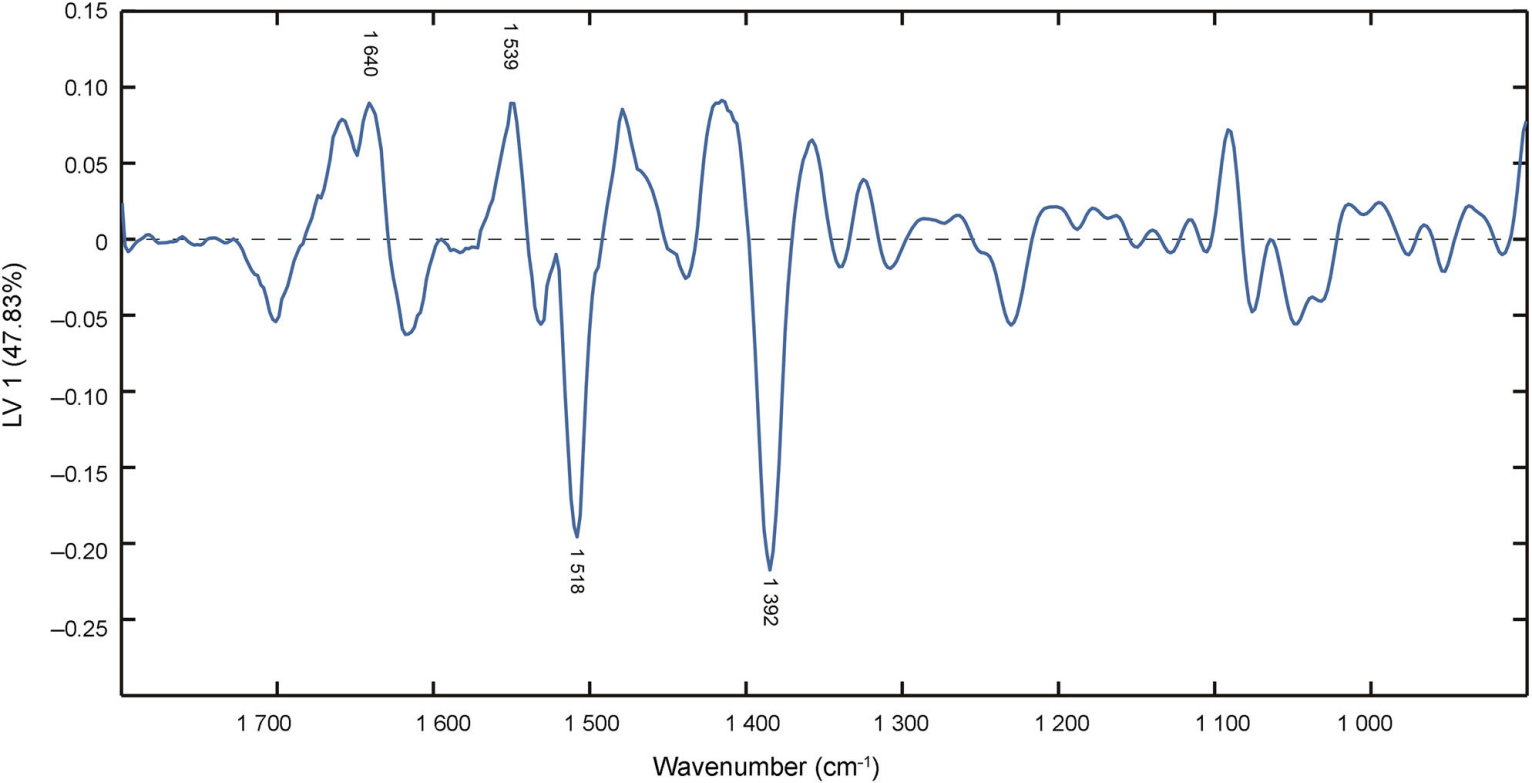
Loading plot of latent variable (LV) 1 in the partial least squares regression (PLSR) model of second derivative transformation. The gray dashed line is the reference line corresponding to the perfect external validation.

**Table 2.** Comparison the partial least squares regression (PLSR) models based on absorbance and second derivative spectra.

Spectral type	LVs	Cross-validation of internal validation			External validation	
		*R*^2^	RMSECV (d)	Explained variance (%)	*R*^2^	RMSEP (d)
Absorbance	6	0.79	0.80	80.91	0.72	1.06
Second derivative	5	0.81	0.77	47.83	0.74	1.02

LVs: latent variables; RMSECV: root mean square error of cross-validation; RMSEP: root mean square error of the predications.

**Figure 6.**
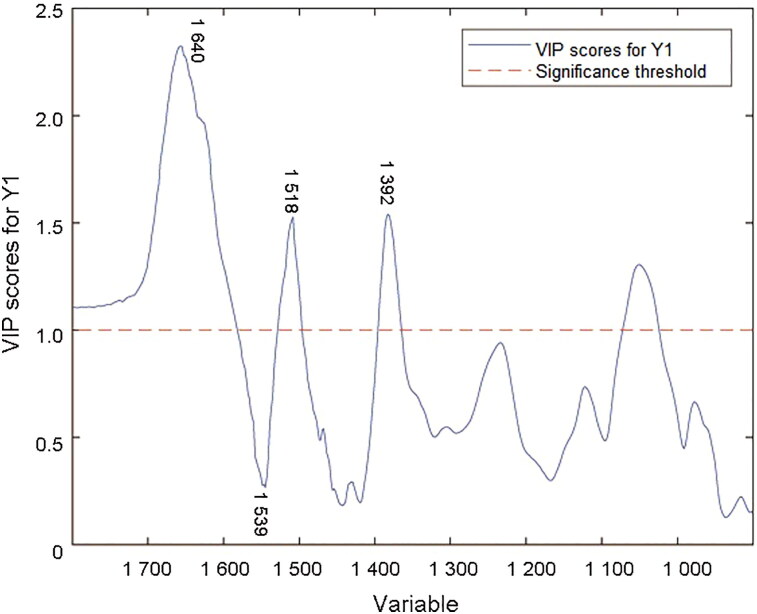
Variable importance in projection (VIP) scores in the partial least squares regression (PLSR) model of second derivative transformation.

According to the loading plot of LV 1 and variable importance in projection (VIP) scores in the PLSR model (Figures 5 and 6), the data correspond with the second derivative spectra. Bands around 1 640 cm^−1^ (amide I bond), 1 539 cm^−1^ (amide II bond) and 1 392 cm^−1^ (due to COO^−^ stretching) show a large proportion of contribution to the establishment of regression model. Tyrosine moieties at approximately 1 518 cm^−1^ also contribute to the PLSR model. The degradations of protein, fructose and other carbohydrates in semen play an important role in the semen stains with aging [21].

Table 2 shows that the model efficiency is influenced by environmental factors, since the environmental conditions of the external validation set were not controlled artificially. Environmental factors will likely lead to more complicated variation.

As a preliminary study, our PLSR models can be considered robust and reliable with the range of human age from 22 to 30 years old. Nevertheless, more work is needed before our approach can be put into real forensic practice. The three substrates used in this study are not the only substrates that appear in crime scenes. Other carriers, such as thick weave fibres, soil and other complex materials, may lead to greater difficulty in detection and age estimation. In addition, there are a number of important factors to consider in future studies, such as increasing the number of samples and the range of age, considering the effects of more environmental factors and contaminations, and combining various developed multivariate analyses.

## Conclusion

This study demonstrates that FTIR, combined with chemometrics, provides an efficient method for estimating the age of human semen stains based on time-dependent changes in the spectra. Based on the results of PCA, the age of semen stains on three different substrates (glass slides, tissues and regenerated cellulose fibres) was predicted over the course of 6 d. For age estimation, PLS of the “bio-fingerprint” spectral region showed satisfactory predictive ability. Additionally, the loading plots of PLS identified sensitive spectral regions of time-dependent changes related to proteins. Thereafter, second derivative transformation improved the efficiency and accuracy of prediction compared to raw absorbance spectral analysis. Ultimately, these results may provide an experimental and theoretical foundation for semen stain age prediction, and therefore great value for future applications in forensic casework.
